# Investigation of some trace elements and hematological and biochemical parameters in the blood of emaciated Omani goats

**DOI:** 10.14202/vetworld.2021.1960-1965

**Published:** 2021-07-29

**Authors:** Turke Shawaf, S. Al Bulushi, M. A. Al-Ali, A. M. A. Meligy, M. Salouci, Jamal Hussen

**Affiliations:** 1 Department of Clinical Sciences, College of Veterinary Medicine, King Faisal University, Al-Ahsa, Saudi Arabia; 2 Department of Animal, Animal Wealth General Directorate, Ministry of Agriculture and Fisheries, Sultanate of Oman; 3 Department of Anatomy, College of Veterinary Medicine, King Faisal University, Al-Ahsa, Saudi Arabia; 4 Department of Microbiology, College of Veterinary Medicine, King Faisal University, Al-Ahsa, Saudi Arabia

**Keywords:** chemistry, emaciated, hematology, Omani goats, serum, trace elements

## Abstract

**Background and Aim:**

The analysis of hematological and biochemical parameters is widely used in assessing animal health status. Limited information is available on trace element levels and their association with hematological and biochemical parameters in Omani goats suffering from emaciation. Therefore, the current study aimed to determine the levels of some trace elements in emaciated Omani goats and their relationship with hematological and biochemical parameters.

**Materials and Methods:**

Goats suffering from emaciation and muscular dystrophy (n=18) were compared with healthy goats (n=12). Venous blood samples for the hematological, biochemical, and trace element analysis were collected from the jugular vein.

**Results:**

Emaciated goats showed significantly lower white blood cell, lymphocyte, and red blood cell counts than the healthy goats. In contrast, the percentages of monocytes and eosinophils were higher in emaciated goats than in healthy ones. In addition, emaciated goats showed higher levels of biochemical parameters alkaline phosphatase, alanine aminotransferase, gamma-glutamyl transferase, aspartate aminotransferase, creatine kinase, and total bilirubin but lower levels of albumin than the healthy goats. The results of trace element analysis revealed lower concentrations of zinc, iron, and selenium in serum from emaciated goats than in serum from healthy goats.

**Conclusion:**

This study identified significant differences in the serum levels of some trace elements and hematological and biochemical parameters between healthy and emaciated Omani goats. The identified differences represent valuable diagnostic biomarkers for the evaluation of the health status of Omani goats.

## Introduction

Goats are economically important animals because they provide high-quality milk and meat [1]. They also have a high survival ability even under severe environmental conditions [2]. Trace elements are vital factors involved in several biological functions, including cell metabolism maintenance, energy production, collagen formation, oxygen transportation, hormone production, enzyme activity, and vitamin synthesis [3-5]. An increase or decrease in trace element levels is usually associated with several detrimental effects, including abnormalities in metabolic, hormonal, immunological, and reproductive functions [4]. However, trace element deficiency is not always associated with clinical signs [6,7]. Selenium (Se) is an important trace element that acts as an antioxidant [8,9] and a cofactor for metalloenzymes [5]. Se deficiency is usually associated with several abnormalities, including placenta retention, impaired fertility, neonatal weakness, and abortion [10]. Acting as an enzymatic and protein cofactor, copper (Cu) plays important roles in the immune system [11]. Iron (Fe) is required for cellular growth, oxygen binding and transportation, and electron transfer [12]. Fe deficiency is associated with different immune system disturbances [13]. Zinc (Zn) is a component of many cellular and nuclear proteins and therefore plays a key role in gene expression regulation [14] and body growth [5].

Although emaciation is not a disease rather a sign associated with many chronic diseases [15,16], it mirrors the pathogenesis and severity of health ^­^disorders [15]. Many factors could be responsible for emaciation and wasting in goats. This mainly includes hunger and poor nutrition [17], parasitic infestations [18], and chronic diseases [19]. In addition, trace element deficiency is one of the major factors that cause wasting in goats [20], resulting in many health disorders with big economic losses [17,19]. The estimation of the normal values of trace elements and their abnormalities is essential tool for veterinary practice in different animal species [21].

As there are only a limited number of studies on trace element evaluation in Omani goats, the current study compared the trace element levels between healthy and emaciated Omani goats and investigated their association with hematological and biochemical parameters.

## Materials and Methods

### Ethical approval

The study was approved by the Ethics Committee at King Faisal University in Saudi Arabia.

### Study period and location

The study was conducted from September 2018 to March 2019 in the Eastern Province of the Kingdom of Saudi Arabia.

### Animals

In the present study, 18 goats (aged between 1 and 3 years) were selected from goats presented with muscle wasting and atrophy at the Veterinary Teaching Hospital, King Faisal University. The diseased animals showed neither hyperthermia nor leukocytosis, indicating the non-infectious nature of the disorders. For comparison, 12 healthy goats from the Research Station at King Faisal University were used as control animals. Body condition scores were used as sampling criteria to differentiate between healthy and emaciated goats based on muscling and fat deposition [22,23]. Emaciated goats had a body condition score of <2 out of 4 total score points. All animals were clinically examined before blood sample collection. The breeding status, animal feed, and source of drinking water were the same in the healthy and affected goats. As there is no pasture feeding in the geographical area where the present study was conducted, all goats from both groups were raised in similar conditions and fed on closely concentrated and filling feeds.

### Blood hematology and biochemistry analysis

Blood samples were collected from the jugular vein into two tubes (Guangzhou Improve Medical, China), one of which contained ethylenediaminetetraacetic acid for blood hematology and the other without anticoagulants for serum separation. All samples were maintained on ice and transferred into the laboratory within 2 h of collection. Blood hematology was conducted using the CELL-DYN 3700 analyzer for total red blood cell (RBC) count, total and differential white blood cell (WBC) count, hemoglobin, hematocrit, mean corpuscular volume, mean corpuscular hemoglobin, mean corpuscular hemoglobin concentration, red cell distribution width, and mean platelet volume. The serum was collected after centrifuging the samples at 1000× *g* for 10 min. Serum biochemistry was analyzed using a VetScan VS2 (ABAXIS, USA) for the mammalian liver profile, which includes the following parameters: Albumin (ALB), alanine aminotransferase (ALT), alkaline phosphatase (ALP), bile acids, cholesterol, gamma-glutamyl transferase (GGT), total bilirubin (TBIL), and blood urea nitrogen.

### Trace element analysis

The levels of the trace elements Fe, Zn, Cu, and Se were estimated using the Shimadzu AA-7000 Atomic Absorption Spectrophotometer (Japan) coupled with Flame Atomic Absorption Spectrometry (FAAS) and a graphite furnace atomic absorption spectrometry system (GFAAS). Furthermore, in FAAS, an air/acetylene gas (10:1.5) was used. Flame atomic absorption was used for the analysis of Cu and Fe, whereas Se analysis was performed through GFAAS using argon (being an inert gas). The samples were injected into the GFAAS and FAAS using the Shimadzu ASO-6100 Automatic Sampler. The digestion of serum samples was done using the Mars Xpress Microwave Digestion System (CEM Cooperation, Mathews, North Carolina, USA). All of the digestion procedures were done using polytetrafluoroethylene vessels. A 5 mL sample of concentrated nitric acid was used to wash the vessels before each digestion process. A quantitative analysis of the samples was performed by external calibration. The serum samples (1 mL) were placed in polytetrafluoroethylene digestion vessels with 5 mL of 65% nitric acid and 3 mL of 30% hydrogen peroxide [24,25]. The digestion process started with the temperature ramped to 125°C within 6 min. Then, the temperature was ramped to 185°C within 15 min followed by a later repeat of 15 min. After 15 min, a cooling system was applied for 5 min. The samples were diluted to 50 mL Milli-Q water (Millipore, Bedford, MA) in 50 mL tubes. After dilution and filtration using Whatman filter paper 1, the digested samples were analyzed by atomic absorption spectrophotometry according to the method described by Pakshirajan *et al*. [26]. Calibration curves were obtained from the stock solutions of Cu, Fe, Zn, and Se standard solution (1000 mg/L) that was purchased from commercial stock solutions (Merck; Darmstadt, Germany). The full quantitative analysis model was used for all measurements [24,25].

### Statistical analysis

The obtained results for blood hematology, biochemical analysis, and trace element analysis were recorded using Microsoft Excel. The statistical analysis, including the calculation of standard error, range, and mean, was performed using GraphPad Prism 7 (GraphPad Software, Inc., San Diego, CA, USA). For the comparison between healthy and affected goats, the Student’s t-test was used. p<0.05 was considered statistically significant.

## Results and Discussion

The assessment of hematological and biochemical parameters is a widely used tool for the evaluation of health status in farm animals [27]. The levels of these parameters could be affected by several physiological and pathological factors, including stress, pregnancy, management, diseases, nutrition, and environmental effects [28]. As there is limited information on trace element levels and their association with hematological and biochemical parameters in emaciated Omani goats, the current study determined the levels of some trace elements in healthy and emaciated Omani goats and investigated their relationship with hematological and biochemical parameters.

Table-1 shows the mean±SD values of the main hematological parameters in the blood of healthy (n=12) and emaciated (n=18) goats. There were significantly fewer WBCs, lymphocytes, and RBCs in the emaciated goats than in the healthy goats. In contrast, the percentages of monocytes and eosinophils (5.4±2.25 and 9.8±4.9, respectively) were higher in the emaciated goats than in the healthy goats. The mean±SD values of serum biochemical parameters for the healthy and emaciated goats are listed in Table-2. For most of the analyzed parameters, a significant increase was observed in the emaciated goats compared with the healthy goats; these include ALP, ALT, GGT, aspartate aminotransferase (AST), creatine kinase (CK), and TBIL. Only the concentration of ALB was lower in the emaciated goats than in the healthy ones.

**Table-1:** Hematological analysis (mean±SD with p values) of healthy and emaciated Omani goats.

Parameter	Healthy (n=12)		Emaciated (n=18)		p-value
	
	Mean±SD	Range	Mean±SD	Range	
WBC (K/micro L)	12.01±1.24	10.7-16.9	9.58±5.11	6.7-17.8	0.021
NEU %	58.48±8.25	51.4-67.8	61.3±13.42	46.39-78.1	0.105
LYM %	34±4.27	29.5-37.8	23.3±10.66	13.5-38.9	0.015
MONO %	1.21±0.44	0.95-2.01	5.4±2.25	1.3-7.9	0.009
EOS %	3.88±0.48	1.66-4.52	9.8±4.9	4.8-15.3	0.02
BASO %	0.50±0.06	0.48-0.61	0.63±0.25	0.22-1.35	0.1
RBC (M/micro L)	11.88±1.56	10.23-14.21	9.5±1.93	7.51-12.65	0.04
HGB (g/dL)	12.11±0.79	11.62-13.43	9.53±1.94	7.25-11.12	0.038
HCT %	37.07±3.12	35.32-42.5	34.56±4.24	30.52-39.77	0.033
MCV (fL)	27.01±2.33	26.12-30.2	28.41±2.52	25.5-31.01	0.32
MCH (pg)	9.65±1.82	7.42-11.13	7.33±2.98	5.47-10.3	0.044
MCHC (g/dL)	28.51±3.07	25.3-31.54	25.33±5.44	20.3-31.7	0.04
RDW %	34.44±2.93	31.8-38.5	33.22±1.87	30.1-36.0	0.66

SD=Standard deviation, WBC=White blood cell, NEU=Neutrophils, LYM=Lymphocyte, MONO=Monocytes, EOS=Eosinophils, BASO=Basophils, RBC=Red blood cell, HGB=Hemoglobin, HCT=Hematocrit, MCV=Mean cell volume, MCH=Mean cell hemoglobin, MCHC=Mean cell hemoglobin concentration, RDW=Red blood cell distribution width

**Table-2:** Levels of some serum biochemical parameters in healthy and emaciated Omani goats.

Parameter	Healthy (n=12)		Emaciated (n=18)		p-value
	
	Mean±SD	Range	Mean±SD	Range	
ALP (IU/L)	68.3±25.08	45-102	87±36.17	45-124	0.022
ALT (IU/L)	25.23±6.31	29.4-31.3	43.37±15.34	29-58.2	0.023
GGT (IU/L)	31.12±11.32	18-43	61.02±31.34	28-98.2	0.008
AST (IU/L)	88.2±29.75	51-111.4	132.1±51.34	61-188.4	0.02
CK (IU/L)	102.4±58.12	55-163.3	188.2±64.4	104-224	0.013
BA (µmol/L)	51.21±34.21	15-88.4	48.4±18.5	29-67	0.32
TBIL (mg/dL)	0.31±0.08	0.2-0.39	0.65±0.1	0.3-0.75	0.01
ALB (g/dL)	3.7±0.37	2.2-4.12	3.01±0.72	2.35-3.88	0.035
BUN (mg/dL)	14.72±5.11	10-20.3	12.98±3.55	9-16.3	0.065
CHOL (mg/dL)	63.12±26.32	21-113	58.5±14.25	39-72	0.08

SD=Standard deviation, ALP=Alkaline phosphatase, ALT=Alkaline phosphatase, GGT=Gamma-glutamyl transferase, AST=Aspartate aminotransferase, CK=Creatine kinase, BA=Bile acid, TBIL=Total bilirubin, ALB=Albumin, BUN=Blood urea nitrogen, CHOL=Cholesterol

**Figure-1:**
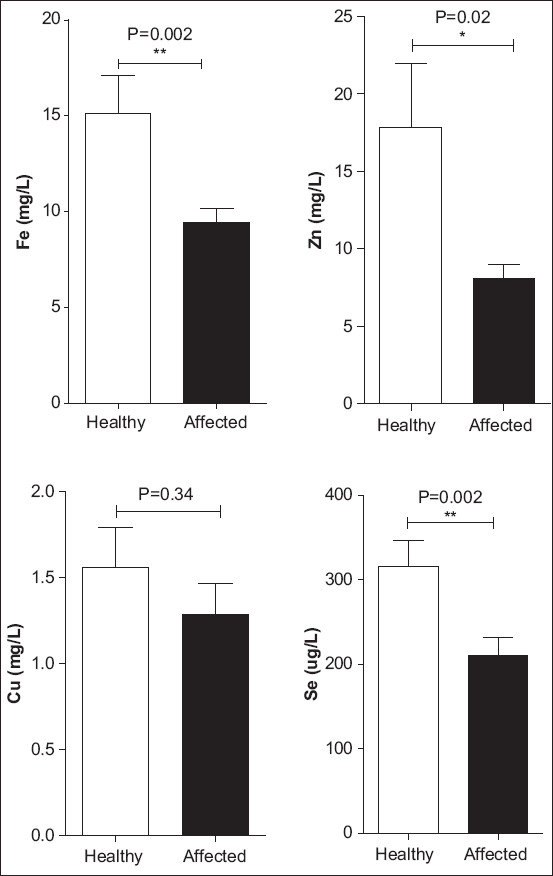
Changes in serum trace elements (iron, zinc, copper, and selenium) levels in healthy and emaciated goats; *p<0.05; **p<0.01 versus controls.

The serum levels of trace elements for both healthy and emaciated Omani goats are presented in Table-3 and Figure-1. The mean concentration of Zn was significantly lower (p=0.02) in the serum of the emaciated Omani goats (8.11±2.44) than in the serum of the healthy goats (17.84±10.89). Similarly, a highly significant decrease in the concentrations of Fe and Se was also observed in the serum of the emaciated goats (Fe=8.7±2.7 and Se=210.4±58.57) compared with that in healthy goats (Fe=15.12±5.28 and Se=316.0±80.27). No significant differences in the serum Cu levels were observed between the two groups of animals.

**Table-3:** Concentration of serum trace elements (iron, zinc, copper, and selenium) in healthy and emaciated Omani goats; *p<0.05; **p<0.01.

Parameter	Healthy (n=12)		Emaciated (n=18)		p-value
	
	Mean±SD	Range	Mean±SD	Range	
Iron (mg/L)	15.12±5.28	10.08-22.85	8.7±2.7	5.45-13.98	0.002
Zinc (mg/L)	17.84±10.89	7.75-37.81	8.11±2.44	3.6-11.78	0.02
Copper (mg/L)	1.64±1.14	0.15-3.44	1.07±0.57	0.52-1.97	0.34
Selenium (µg/L)	316±80.27	229.7-473.3	210.4±58.57	127.5-333.2	0.002

SD=Standard deviation

The observed decrease in the WBC count in the present study was reported in goats and sheep affected with internal parasites with signs of muscle atrophy [28,29]. The present study showed that the emaciated goats with lower WBC counts had significant Fe, Zn, and Cu deficiencies, which contrasts with the results of the previous studies [20] that failed to identify any relationship between mineral deficiency and WBC counts in goats. The higher percentage of monocytes and eosinophils in the emaciated goats in the present study is in agreement with the eosinophilia and monocytosis reported in animals with muscle dystrophy [30,31].

The decreased RBC count observed in the emaciated goats in the present study is in line with the previous reports [32]. The decrease in the RBC count could be associated with Se deficiency, as Se contributes to the synthesis of the cell membrane of RBC [33]. On the other hand, the lower RBC count and the reduced concentration of hemoglobin in the emaciated goats relative to the healthy goats in the present study could have resulted from malnutrition, as reported by the previous studies [20,32,34].

In the present study, a marked increase in ALP, ALT, GGT, AST, and TBIL was observed in the emaciated goats relative to the healthy ones. Higher activities of hepatic enzymes were reported in emaciated animals with anemia [35,36]. In addition, a similar increase in hepatic enzymes was reported in goats infected with internal parasites [37,38]. A relationship between the elevated serum levels of ALP, AST, and ALT and the imbalance in the trace element levels in goats was found to be associated with increased cell damage [39,40]. Similarly, the increased levels of AST and CK in the affected goats relative to the healthy ones might indicate liver [33] or muscle damage [32].

Fe is a vital element in the production of hemoglobin and myoglobin, with an essential contribution to the activity of catalase, peroxidase, and cytochrome [32,40]. The decreased level of Fe and the positive correlation between Fe concentration and RBC counts in the emaciated goats from the present study are in agreement with the previously reported Fe deficiency in goats [41] or other ruminants [40,42] affected with anemia.

The decreased level of Zn in the serum of the emaciated goats relative to that of the healthy ones is in line with previous reports on emaciated goats [39] or other animals experiencing anorexia [43].

Cu plays an important role in the synthesis of various enzymes, including cytochrome oxidase, ceruloplasmin, and superoxide dismutase [40]. The relationship between Cu deficiency and RBC counts in the emaciated goats in the present study could be explained by the role of Cu in RBC formation, as Cu is found in the transferase enzyme that transfers Fe from the digestive tract through the liver to the bone marrow for hemoglobin formation [32,44]. Cu deficiency was reported in many disorders, including anemia, severe diarrhea, fragile bones, and suppressed growth [34,45].

Se is an essential element with antimutagenic, antioxidative, and antiviral properties [46-48]. Se deficiency leads to compromised immune function, weight loss, weakness, white muscle disease, and diarrhea [46,49]. In the current study, lower Se levels were observed in the emaciated goats relative to the healthy ones, which is similar to the findings reported by Ghanem *et al*. [32]. Se deficiency can cause hemolysis and anemia, as it mainly contributes to RBC protection from free radicals [32]. In agreement with our findings, the previous studies reported significant associations between Se deficiency and elevated AST, ALP, and CK enzyme activities in the serum of animals suffering from emaciation and muscle atrophy [45,50,51].

## Conclusion

The present study identified significant differences in the serum levels of some trace elements and hematological and biochemical parameters between healthy and emaciated Omani goats. The identified normal values of the analyzed parameters support the establishment of reference ranges for Omani goats. In addition, the identified differences represent valuable biomarkers, which could facilitate early diagnosis and the evaluation of the health status of diseased Omani goats.

## Authors’ Contributions

TS, SA, AMAM, and JH: Designed the experiments. TS, SA, AMAM, MS, and MAA: Executed the experiments and analyzed the samples. TS, SA, and JH: Interpreted the data and drafted the manuscript. All authors critically revised, read, and approved the final manuscript.
